# Performance of Bio-Based Foam Packaging for Frozen Fried Chicken Storage

**DOI:** 10.3390/foods15020242

**Published:** 2026-01-09

**Authors:** HyeRyeong Choi, Anuja P. Rananavare, Youn Suk Lee

**Affiliations:** Department of Packaging and Logistics, Yonsei University, Wonju 26393, Gangwon-do, Republic of Korea

**Keywords:** eco-friendly, biofoam, frozen fried chicken storage, lipid oxidation, packaging

## Abstract

Structural and physicochemical deterioration in frozen foods is largely driven by ice crystal formation and growth during storage. Although biofoams offer sustainable alternatives to plastic packaging, bio-based systems designed to mitigate ice crystal-induced quality loss remain limited. In this study, a sodium alginate-based biofoam was synthesized via a facile one-pot method and evaluated for frozen fried chicken packaging. Its moisture, mechanical, and optical properties were compared with those of conventional plastic and paper packaging. The quality of frozen fried chicken was assessed in terms of moisture absorption, color, texture, pH, lipid oxidation (TBARs), and the overall appearance under different freezing conditions. The alginate biofoam exhibited exceptionally high moisture absorption (>2400%) due to its porous and hydrophilic structure, enabling effective moisture management during frozen storage. Samples packaged with the biofoam showed reduced moisture loss, lower lipid oxidation, and improved color and surface texture stability compared with conventional packaging, particularly under freeze–thaw conditions. These findings demonstrate that sodium alginate-based biofoam is a promising eco-friendly packaging material for maintaining the physicochemical quality of frozen ready-to-eat foods.

## 1. Introduction

The quality of frozen foods is strongly influenced by structural and physicochemical changes that occur during freezing and frozen storage. In particular, the formation, size, and distribution of ice crystals play a critical role in determining textural integrity, water-holding capacity, and overall sensory attributes of food products. Large or unevenly distributed ice crystals can damage cellular structures through mechanical disruption and osmotic stress arising from solute concentration gradients, leading to moisture loss, texture deterioration, and reduced consumer acceptance during prolonged frozen storage [1,2,3,4]. Frozen foods are widely consumed because they offer extended shelf life while retaining much of the original flavor, texture, and nutritional quality of fresh products. However, maintaining these qualities remains challenging, as freezing fundamentally alters food microstructure through the phase transition of water to ice. Previous studies have primarily focused on controlling frozen food quality through the optimization of freezing rate, formulation, or processing conditions. Rapid freezing, for example, is known to produce smaller ice crystals and reduce structural damage compared to slow freezing [5]. Similarly, ingredient composition and processing strategies have been explored to improve the quality of frozen noodles, pasta, and other convenience foods [6,7,8]. Despite these advances, strategies that address frozen food quality from a packaging material perspective remain relatively underexplored.

Packaging plays a crucial yet often overlooked role in frozen food preservation by influencing heat transfer, moisture migration, condensation, and temperature fluctuations during storage. Inadequate packaging can promote surface dehydration, condensation–evaporation cycles, and ice recrystallization, all of which accelerate quality degradation. Therefore, developing packaging systems that can actively regulate moisture and provide thermal insulation represents a promising approach to mitigating freezing-induced damage and preserving food quality during long-term frozen storage. In this context, bio-based foams have gained increasing attention as sustainable alternatives to petroleum-derived foams owing to their biodegradability, renewability, and alignment with circular economy principles. Biofoams fabricated from natural polymers such as starch [9,10] cellulose [11], and alginate have demonstrated mechanical and thermal properties comparable to those of conventional synthetic foams. Notably, eco-friendly foams derived from agricultural byproducts and polysaccharides have shown potential to replace plastic packaging materials while reducing environmental impact [12].

Sodium alginate (SA), a naturally occurring polysaccharide extracted from brown seaweed, is particularly attractive for biofoam fabrication due to its excellent hydrophilicity, biocompatibility, and environmentally benign nature [13]. The intrinsic viscosity, gel-forming ability, and air entrapment capacity of SA facilitate the formation of lightweight, highly porous foams with low thermal conductivity [14,15]. Previous studies have reported SA-based foams with porosities exceeding 90% and ultralow densities comparable to commercial polymer foams [16]. Moreover, the abundant hydroxyl and carboxyl groups in SA impart strong water-binding capability through hydrogen bonding, enabling effective moisture absorption and retention [17,18,19]. These characteristics suggest that alginate-based biofoams may function not only as passive packaging materials but also as active moisture-regulating and insulating systems during frozen storage. By absorbing surface moisture and limiting condensation within the packaging environment, SA foams have the potential to reduce dehydration, suppress ice recrystallization, and mitigate freezer burn. Such moisture regulation is particularly important for frozen foods, where uncontrolled water migration often leads to the formation of large ice crystals that compromise texture and appearance [20,21].

Despite these advantages, few studies have systematically investigated bio-based foam packaging as a strategy to inhibit ice crystal growth and moisture migration during frozen storage. In particular, the application of alginate-based biofoams for frozen food packaging remains largely unexplored. Therefore, the present study aims to address this research gap by developing a sodium alginate-based biofoam and evaluating its ability to inhibit ice formation during freezing, in comparison with commercially available plastic and paper packaging materials under different temperature conditions. Frozen fried chicken was selected as the model food system because of its high moisture and lipid content, making it especially susceptible to freezing-induced quality deterioration, including texture degradation, moisture loss, and oxidative changes. The biofoam was fabricated using a foaming technique incorporating SA and glycerol as a plasticizer, calcium lauryl sulfate as a foaming agent, and calcium chloride as a cross-linking agent. The effects of packaging and freezing conditions on food quality were evaluated through analysis of density, moisture absorption, color, texture, pH, thiobarbituric acid reactive substances (TBARs), and overall appearance. The findings of this study demonstrate the potential of alginate-based biofoams as a novel, sustainable packaging solution for improving frozen food quality during storage.

## 2. Materials and Methods

### 2.1. Chemicals

Sodium alginate (SA; purity 99%, molecular weight 32–250 kDa) was purchased from Duksan Pure Chemicals Co., Ltd. (Ansan, Gyeonggi-do, Republic of Korea). Calcium carbonate (CaCO_3_, 98.5%) and D-glucono-δ-lactone (GDL, 99%) were obtained from Daejung Chemicals and Metals Co., Ltd. (Incheon, Gyeonggi-do, Republic of Korea) and used as components of an internal gelation system. Glycerol (99%) was used as a plasticizer, and sodium dodecyl sulfate (SDS, 97%) was employed as a foaming agent. Commercially available plastic pouches (CPs) and paper-based pouches (PPs) were obtained from Daesang Co. (Seoul, Republic of Korea) and used as reference packaging materials.

### 2.2. Preparation of Sodium Alginate Biofoam

SA was dissolved in distilled water at a concentration of 1.25% (*w*/*v*), selected based on preliminary trials and the literature reports [13] indicating that this concentration provides sufficient solution viscosity for stable foam formation while maintaining processability. Glycerol was added at 7.5% (*w*/*v*) relative to the total solution volume to improve flexibility and reduce brittleness of the dried foam, as commonly reported for polysaccharide-based foams. The solution was mixed at 2000 rpm for 1 min to ensure homogeneity. SDS was incorporated at 0.09% (*w*/*v*) as a surfactant to reduce surface tension and promote air incorporation during foaming. This concentration was chosen based on preliminary optimization to achieve stable foam structure without excessive collapse. The mixture was subsequently whipped at 4000 rpm for 5 min to generate a uniform wet foam. Internal ionic cross-linking of alginate was achieved by the successive addition of GDL and CaCO_3_, each at 30% (*w*/*v*) relative to the dry weight of SA. In this system, GDL gradually hydrolyzes to gluconic acid, leading to a controlled decrease in pH, which in turn triggers the slow dissolution of CaCO_3_ and the release of Ca^2+^ ions. These calcium ions interact with the guluronic acid blocks of alginate to form an ionically cross-linked network, thereby stabilizing the foam structure during drying. The wet foam was cast into square molds (12.5 × 12.5 cm) and dried in a convection oven at 50 °C for 22 h until constant weight was achieved. The final dried biofoam had an average thickness of 1 ± 0.2 cm, density of 12.83–108.39 mg/cm^3^, and porosity of 90.43–97.12%, as determined by gravimetric and dimensional analyses.

### 2.3. Experimental Conditions

For frozen storage experiments, the following three packaging systems were evaluated: (i) sodium alginate biofoam placed inside a plastic pouch (BP), (ii) a commercially available plastic pouch (CP), and a commercially available paper pouch (PP). The commercial plastic pouches were composed of multilayer low-density polyethylene-based film with an average thickness of 100 μm, while the paper pouches consisted of food-grade paper with an average thickness of 120 μm. All packaging materials were cut to identical dimensions to ensure comparable sample loading and surface area exposure. Frozen fried chicken samples of uniform size and weight were individually packaged using each packaging system. All samples were prepared simultaneously and stored under identical conditions to minimize variability.

**Figure 1:**
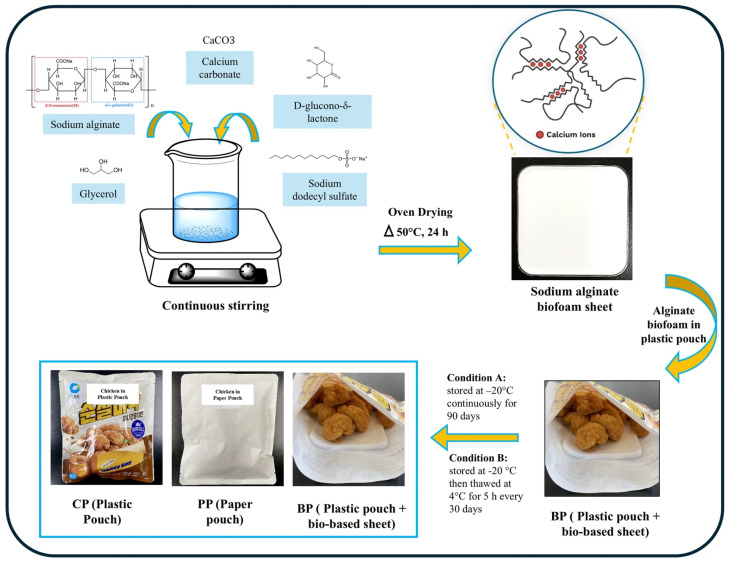
Schematic showing the one-step synthesis of sodium alginate foam and quality evaluation of frozen fried chicken food in different packaging materials.

Two storage conditions were employed to evaluate packaging performance during frozen storage (Figure 1). In condition A (continuous freezing), samples were stored at −20 °C for 90 days without interruption and analyzed at the end of the storage period. In condition B (repeated freeze–thaw cycles), samples underwent freeze–thaw treatments at 30-day intervals (0, 30, 60, and 90 days). Each freeze–thaw cycle consisted of thawing at 4 °C for 5 h, followed by storage at 4 °C for 24 h prior to analysis, after which samples were returned to −20 °C. The 30-day interval was selected to simulate periodic temperature fluctuations commonly encountered during long-term frozen storage, transportation, and retail handling. Prior to physicochemical analysis, all frozen samples were thawed at 4 °C for 3 h to ensure uniform handling conditions. For each packaging system and storage condition, a minimum of n = 3 independent samples were analyzed, and all measurements were performed in triplicate, unless otherwise stated. This experimental design enabled direct comparison of the effects of packaging material and freezing history on moisture migration, ice recrystallization-related quality changes, and overall stability of frozen fried chicken.

### 2.4. Performance Evaluation of Packaging Materials

The packaging materials were evaluated according to their water absorption, water vapor permeability, surface morphology, and mechanical properties during storage. Prior to these analyses, the packaging materials were pretreated using the Gelbo flex durability test to simulate the mechanical stress of handling and storage during transportation and distribution. Each packaging sample was cut into pieces (200 × 280 mm^2^) and tested using a Gelbo Flexing Tester (QM650G, QMESYS, Seoul, Republic of Korea), according to ASTM F392 standard [22]. The test conditions were as follows: rotation speed, 45 cycles/min; twist angle, 440°; stroke length, 155 mm; and total number of cycles, 35.

First, the moisture absorption properties of packaging samples were evaluated according to the ASTM D570 standard [23]. Bar-shaped samples (76.2 × 25.4 mm^2^) were immersed in 100 mL of water for 24 h. After immersion, the surface moisture was removed and the wet weight was recorded. The moisture absorption (%) was calculated using the following equation:

(1) $$Moisture\;absoption\;\left(\%\right)=\;\left(\frac{\left(W_{1}-W_{0}\right)}{\left(W_{0}\right)}\right)\times 100$$

Second, the water vapor transmission rate (WVTR) of packaging samples was determined using a Water Vapor Transmission Rate Testing System (PERMATRAN-W Model 3/33, Mocon Inc., Minneapolis, MN, USA), according to the ASTM F1249 standard [24], at 37.5 °C, 100% relative humidity, and a nitrogen flow rate of 10 cm^3^/min.

Third, the surface morphology of the packaging materials was observed using an optical microscope (Xi-cam; Bestecvision, Gyeonggi, Republic of Korea).

Fourth, the tensile strength, elastic modulus, and elongation at break of packaging samples were measured using a universal testing machine (QM 100T, QMESYS, Republic of Korea), in accordance with the ISO 527-3 standard [25]. Packaging samples were cut into strips (115 × 25 mm^2^) and tested at a crosshead speed of 100 mm/min.

### 2.5. Quality Evaluation of Frozen Foods

Although the number of quality indicators evaluated in this study are limited, the selected parameters—moisture behavior, lipid oxidation (TBARs), color, texture, pH, and the overall appearance—are widely recognized as key physicochemical indicators of quality deterioration in frozen meat and fried food products. These indicators collectively reflect water migration, oxidative stability, structural integrity, and visual quality, which are the primary factors governing consumer acceptability during frozen storage.

#### 2.5.1. Moisture Content

The moisture content of frozen fried chicken samples was determined following the AOAC (1990) [26] atmospheric heating and drying method. Approximately 5 g of thawed sample was oven-dried (OF-12GW, JEIOTECH Co., Ltd., Seoul, Republic of Korea) at 105 °C for 12 h or until a constant weight was obtained. The moisture content was calculated using the following equation:

(2) $$Moisture\;content\;\left(\%\right)=\;\left(\frac{\left(W_{1}-W_{2}\right)}{\left(W_{1}-W_{0}\right)}\right)\times 100$$

where *W*_0_ is the weight of the weighing dish (g), *W*_1_ is the weight of the dish and sample before drying (g), and *W*_2_ is the weight after drying (g). Both the skin and boneless portions of the frozen fried chicken were used, and all measurements were performed in triplicate.

#### 2.5.2. Color

The color of each frozen food sample was determined using a colorimeter (CR-10; Minolta Co., Tokyo, Japan). Hunter color values L* (lightness), a* (+a = red, −a = green), and b* (+*b* = yellow, −*b* = blue) were measured, and the average values were reported. For frozen chicken, samples of similar size were selected, and measurements were performed on flat surface areas. All measurements were repeated 10 times. The total color difference (Δ*E*) of samples before and after storage under condition A or B was calculated using the following equation:

(3) $$\Delta E=\sqrt{{\left(L^{*}-L\right)}^{2}+{\left(a^{*}-a\right)}^{2}+{\left(b^{*}-b\right)}^{2}}$$

where Δ*E* is the total color difference, L* is the lightness, a* is redness (from green to red), and b* is yellowness (from blue to yellow).

#### 2.5.3. Appearance

Changes in the surface quality (such as ice crystal formation) and shape retention of frozen products during the freeze–thaw process were visually evaluated. Photographic images of the samples were obtained at each storage interval for comparison.

#### 2.5.4. Texture Profile

Textural properties, including hardness, springiness, gumminess, and chewiness, were analyzed using a texture analyzer (Ametek TA1 Series; AMETEK Measurement and Calibration Technologies, Wilmington, DE, USA). The frozen chicken samples were cut into cubes (25 × 25 × 25 mm^3^) before testing. The test conditions were set to a test speed of 1.0 mm/s, a compression strain of 50%, and 30 mm. Each test was performed 10 times.

#### 2.5.5. pH

After thawing, 5 g of each frozen fried chicken sample was mixed with 45 mL of distilled water (1:9 ratio) and homogenized at 8000 rpm for 1 min. The pH was measured using a pH meter (Orion 4-Star Plus pH/dissolved Oxygen Benchtop Multiparameter Meter, Thermo Scientific, Waltham, MA, USA). Measurements were taken from both skin and boneless meat. All measurements were performed in triplicate.

#### 2.5.6. Lipid Oxidation (TBARs)

Lipid oxidation was analyzed using the modified method of Witte et al. (1970) [27]. One gram of each frozen fried chicken sample was homogenized in 9 mL of 11% trichloroacetic acid solution and centrifuged at 12,000 rpm for 12 min. The supernatant was filtered through Whatman No. 1 filter paper and diluted to 10 mL with distilled water. Two milliliters of the filtrate were mixed with 2 mL of 0.02 M thiobarbituric acid solution, heated in a water bath at 95 °C for 30 min, and then cooled for 15 min. The absorbance was measured at 532 nm using a UV–Vis spectrophotometer (GENESYS 150, Thermo Fisher Scientific Inc., Waltham, MA, USA).

### 2.6. Statistical Analysis

All experimental data were statistically analyzed using SPSS software (version 28.0; SPSS Inc., Chicago, IL, USA). Prior to analysis, the assumptions of normality and homogeneity of variance were evaluated. Data normality was assessed using the Shapiro–Wilk test, while homogeneity of variances was examined using Levene’s test. As all datasets satisfied these assumptions (*p* > 0.05), one-way analysis of variance (ANOVA) was applied to determine significant differences among mean values. Duncan’s multiple range test was used for post hoc comparisons at a significance level of *p* < 0.05.

## 3. Results and Discussion

### 3.1. Packaging Material Performance

#### 3.1.1. Moisture Absorption

The moisture absorption values of the plastic pouch (CP), paper pouch (PP), and biofoam in a plastic pouch (BP) are presented in Table 1. CP exhibited excellent moisture resistance throughout storage and mechanical stress testing. Owing to its multilayer polymeric structure, CP maintained 0% moisture absorption both before and after the Gelbo flex test, confirming its high structural integrity and effective water vapor barrier performance. In contrast, PP showed a marked increase in moisture absorption after the Gelbo flex test, reaching 14.39%. This increase is attributed to the weakening of fiber–fiber bonding and the formation of microcracks within the paper matrix under repeated mechanical deformation, which compromised its barrier properties.

BP exhibited an exceptionally high moisture absorption rate (>2400%) prior to Gelbo flex testing, reflecting its strong hygroscopic character. This behavior is expected for alginate-based biofoams due to their highly porous architecture and intrinsically hydrophilic polymer network rich in hydroxyl and carboxyl functional groups. Such high moisture uptake may contribute to condensation control within the package by absorbing excess surface moisture, potentially reducing drip loss during thawing and limiting ice recrystallization under freeze–thaw conditions. Although this high absorption capacity limits the applicability of BP as a primary barrier material, it underscores its potential as a functional moisture-absorbing or humidity-regulating component in active or intelligent packaging systems.

Post-Gelbo flex moisture absorption was not measured for BP because the Gelbo flex pretreatment rendered reliable post-test evaluation impractical. The Gelbo flex test was not applied to the sodium alginate biofoam samples due to the brittle and highly porous nature of the dried structure, which is incompatible with repetitive twisting and flexing. Preliminary testing resulted in structural fracture rather than controlled flexural deformation, which does not realistically represent the intended cushioning and moisture-regulating function of the biofoam. Therefore, post-Gelbo flex evaluations were conducted only for CP and PP. This material-specific limitation is acknowledged and discussed accordingly.

#### 3.1.2. Water Vapor Permeability Properties (WVTRs)

After pretreatment with the Gelbo flex test, the paper pouch (PP) exhibited the greatest increase in water vapor transmission rate (WVTR) during frozen storage, rising from 8.08 ± 0.23 g/m^2^·day at day 0 to 20.07 ± 0.27 g/m^2^·day after 90 days, corresponding to an increase of approximately 148.4% (Table 2). This pronounced increase indicates poor moisture barrier stability and can be attributed to the fibrous and porous structure of paper, which becomes increasingly susceptible to moisture permeation following mechanical deformation. Gelbo flex pretreatment likely induced microcracks and disrupted fiber–fiber bonding, thereby facilitating water vapor diffusion. These effects were further exacerbated during frozen storage by ice crystal formation and repeated moisture migration. In contrast, the plastic pouch (CP) exhibited a comparatively lower increase in WVTR, from 4.25 ± 0.04 to 9.04 ± 0.03 g/m^2^·day (112.7% increase), indicating greater resistance to moisture transfer under identical storage conditions. This behavior is primarily attributed to the dense and continuous polymeric matrix of CP, which effectively limits water vapor diffusion even after mechanical stress. Although a gradual increase in WVTR was observed, likely due to minor structural fatigue induced by Gelbo flex testing and prolonged frozen storage, CP maintained relatively stable barrier performance compared with PP.

These results demonstrate that material microstructure and resistance to mechanical damage are critical factors governing WVTR behavior during frozen storage. Elevated WVTR in PP is associated with increased moisture migration, condensation, and drip loss during thawing, whereas the lower WVTR observed for CP contributes to improved moisture retention and quality stability of frozen foods. The mechanical properties of the sodium alginate-based biofoam were not quantitatively evaluated in this study. Owing to its highly porous and brittle structure in the dried state, conventional mechanical testing methods commonly applied to flexible packaging materials were not applicable. Consequently, the biofoam was assessed primarily for its moisture absorption behavior and functional performance as a cushioning and humidity-regulating component. Future studies should employ tailored mechanical characterization approaches to better evaluate its structural performance under application-relevant conditions.

#### 3.1.3. Surface Morphology

**Figure 2:**
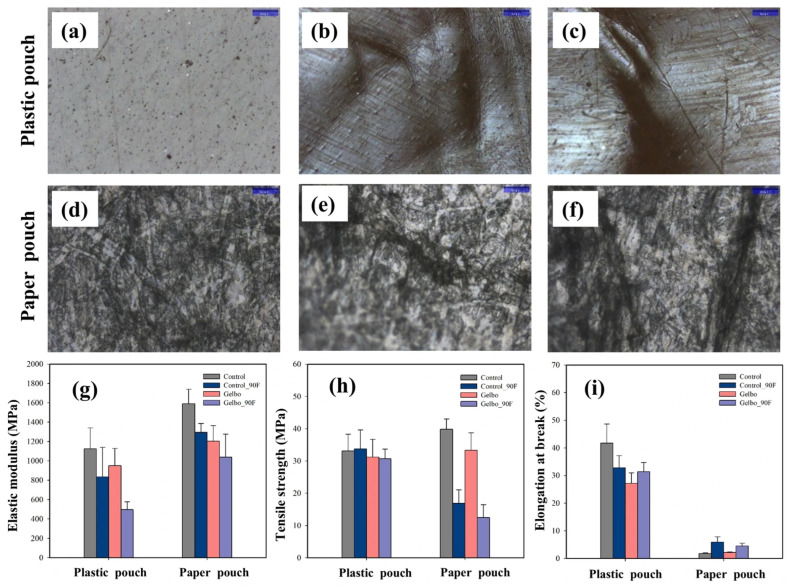
Surface morphology and mechanical properties of plastic and paper pouches under Gelbo flex testing and frozen storage. (**a**–**f**) Optical microscope images of the plastic pouch (**a**–**c**) and paper pouch (**d**–**f**) before the Gelbo flex test (**a**,**d**), after the Gelbo flex test (**b**,**e**), and after the Gelbo flex test followed by 90 days of frozen storage (**c**,**f**). (**g**–**i**) Mechanical properties of plastic and paper pouches under the following different conditions: (**g**) elastic modulus, (**h**) tensile strength, and (**i**) elongation at break. Control: before Gelbo flex test; Gelbo: after Gelbo flex test; and 90F: after 90 days of frozen storage.

Figure 2a–f present optical microscopy images of PP and CP before and after the Gelbo flex test and after 90 days of storage. CP exhibited minimal surface damage after the Gelbo flex test and 90 days of frozen storage, maintaining a stable structure (as evidence by excellent water resistance) without the formation of pores or cracks. This indicates that the multilayer polymer structure effectively preserved its mechanical integrity under repeated flexing stress. In contrast, PP showed severe cracking and surface rupture following the same test. This deterioration of the paper composite layer was evidenced by the marked increase in both moisture absorption and WVTR, revealing structural weakness and reduced barrier performance.

#### 3.1.4. Mechanical Properties

Elastic modulus, tensile strength, and elongation of CP and PP are shown in Figure 2g–i, respectively. Exposure to Gelbo flex testing and frozen storage reduced the elastic modulus and tensile strength of both materials. However, CP exhibited the highest structural stability, retaining 92.7% of its original tensile strength. In contrast, PP showed the greatest reduction in mechanical stability, preserving only 68.6% of its initial tensile strength. These results indicate that CP is more resistant to low temperatures and mechanical stress than PP. The mechanical properties of the sodium alginate-based biofoam were not evaluated in this study because its highly porous and brittle-dried structure was incompatible with standard tensile and flexural testing methods. Under mechanical loading, the biofoam fractured prematurely, preventing reliable measurement of elastic modulus, tensile strength, and elongation.

### 3.2. Frozen Food Quality

#### 3.2.1. Moisture Content, pH, and Lipid Oxidation

**Figure 3:**
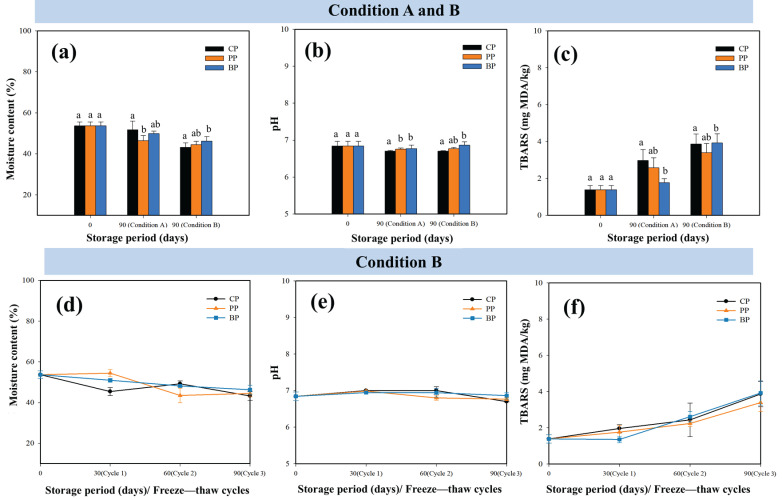
Moisture content, pH, and thiobarbituric acid reactive substance (TBAR) values of fried chicken under different frozen storage conditions as follows: (**a**–**c**) condition A (continuous freezing). Different letters (a, b, and ab) above the bars indicate significant differences among treatments at the same storage period (*p* < 0.05) and condition B (repeated freeze–thaw cycles). (**d**–**f**) Changes in moisture content, pH, and TBAR values under condition B. Bars sharing the same letter are not significantly different (*p* > 0.05).

The moisture content (reported on a wet basis), pH, and lipid oxidation levels (TBARs) of frozen fried chicken under conditions A and B are presented in Figure 3. Figure 3d–f detail the incremental quality changes associated with freeze–thaw stress, which was applied on days 0, 30, 60, and 90 during the 90-day frozen storage period (condition B).

Food samples stored under condition B exhibited a slightly lower moisture content than those stored under condition (Figure 3a), reflecting higher moisture migration and drip loss caused by repeated freeze–thaw stress [28]. Among the three packaging types, BP showed marginally increased moisture retention under both conditions; however, the differences between CP, PP, and BP were not statistically significant (*p* > 0.05). The pH of all food samples remained stable within a narrow range (6.8–7.0) throughout the 90-day storage period (Figure 3b), with no statistically significant differences between packaging types or storage conditions (*p* > 0.05), indicating that neither storage nor packaging notably affected chemical stability. The marginally higher pH stability observed for BP may be attributed to its bio-based composition, which can help regulate moisture and reduce oxidative reactions. These findings indicate that the intrinsic product composition and matrix structure play a more dominant role in maintaining chemical equilibrium during frozen food storage. Average TBAR values, expressed as milligrams of malondialdehyde per kilogram of sample (mg MDA/kg), for food samples stored in the three packaging types increased from approximately 2.5 mg MDA/kg under condition A to approximately 3.6 mg MDA/kg under condition B (Figure 3c), reflecting enhanced lipid oxidation caused by repeated freeze–thaw stress. This increase can be attributed to the oxidative reactions triggered by membrane disruption and oxygen exposure during thawing [29]. Although BP samples exhibited slightly lower TBAR values under condition A and PP performed marginally better under condition B, these differences were not statistically significant (*p* > 0.05). The absence of significant differences among packaging materials between the two conditions indicates that all tested materials provided comparable oxidative stability, supporting the suitability of BP as an effective alternative to conventional packaging for frozen food preservation.

Under condition B, freeze–thaw cycles were conducted on days 0, 30, 60, and 90. During this process, the moisture content of frozen fried chicken gradually decreased from approximately 53% at day 0 (Figure 3d), which can be attributed to ice crystal formation and thaw-induced structural damage that disrupted cellular integrity and promoted water migration. Among the packaging materials, PP exhibited slightly lower moisture retention between days 30 and 60, likely owing to the deformation of its cellulose fiber matrix and reduced water-holding capacity. Noticeable moisture loss occurred under condition B, indicating that the intrinsic composition and structural matrix of fried chicken have a greater influence on moisture retention than the type of packaging material used during repeated freeze–thaw stress. The pH of frozen fried chicken remained relatively stable throughout condition B (Figure 3e), with a slight increase up to day 60, followed by a minor decline up to day 90, which may be associated with protein denaturation and the release of basic nitrogenous compounds during repeated freezing and thawing [30]. However, the magnitude of this change was small, and no statistically significant differences were observed among packaging types (*p* > 0.05). These results confirm the comparable chemical stability of packaging materials under repeated freeze–thaw cycling and the greater role of product composition and matrix structure. The TBAR values of frozen fried chicken increased gradually over the 90 days of repeated freeze–thaw cycles (Figure 3f), indicating progressive lipid oxidation. The most pronounced TBAR increase occurred at 90 days, likely because of the high fat content of fried chicken and the combined effects of frying and repeated freeze–thaw stress, which enhance oxidative degradation through membrane disruption and oxygen exposure [31]. Despite this increase, no statistically significant differences were observed between packaging types (*p* > 0.05), suggesting similar levels of oxidative protection. These results imply that lipid oxidation under condition B was governed by the intrinsic properties of the product, i.e., the fat-rich matrix of fried chicken. The comparable TBAR values across packaging types suggested equivalent protection against lipid oxidation during repeated freeze–thaw storage. The observed trends in moisture content and TBAR values suggest a close relationship between moisture loss and lipid oxidation during frozen storage. Repeated freeze–thaw cycles promoted moisture migration and structural damage, which increased lipid exposure to oxygen and consequently accelerated oxidative reactions. Samples experiencing greater moisture loss generally exhibited higher TBAR values, particularly under condition B, confirming that effective moisture management plays a critical role in limiting lipid oxidation in frozen fried chicken. Therefore, packaging systems that help regulate moisture and reduce surface dehydration, such as BP, can indirectly contribute to improved oxidative stability during frozen storage.

#### 3.2.2. Food Appearance and Ice/Frost Formation

**Figure 4:**
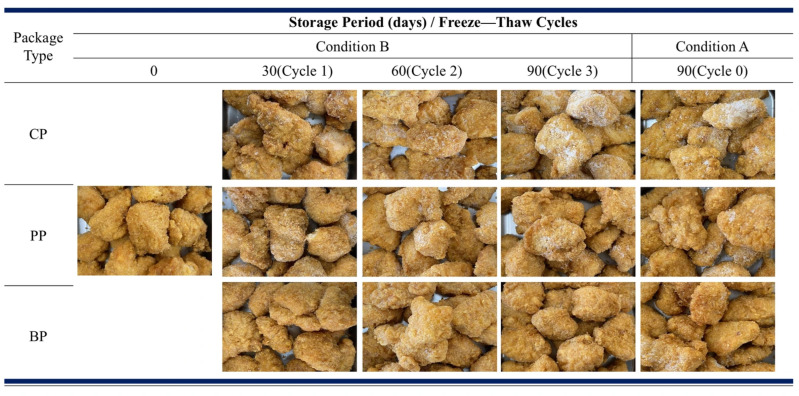
Visual appearance of fried chicken packaged in a plastic pouch (CP), paper pouch (PP), or biofoam within a plastic pouch (BP) after storage under condition A (continuous freezing) and condition B (repeated freeze–thaw cycles).

Figure 4 shows the visual appearance of frozen fried chicken packaged with CP, PP, and BP materials under storage conditions A and B. The formation of ice crystals and surface frost increased with storage time under both conditions in the following order: CP > PP > BP. Fried chicken packaged in CP showed more pronounced frost and crystal formation; this was attributed to the high moisture and oxygen barrier properties of CP, which tend to trap internal moisture that likely condenses and recrystallizes during repeated freezing and thawing cycles, leading to visible frost accumulation. In contrast, PP exhibited relatively fewer ice crystals and less frost formation [32]. This behavior may be due to the moisture-buffering capacity of the cellulose-based paper layer, which allows gradual moisture release and prevents excessive condensation within the package [33]. BP demonstrated the least frost and ice crystal formation, maintained stable internal humidity, and minimized condensation. This was attributed to the superior moisture regulation of BP, which likely contributed to better visual preservation and reduced freezer burns throughout storage. Thus, the type of packaging material influenced frost and ice crystal formation, with BP providing the most effective moisture control under both continuous freezing and repeated freeze—thaw conditions.

#### 3.2.3. Food Color

The color parameters (L*, a*, b*, and ΔE) of frozen fried chicken packaged in CP, PP, and BP under storage conditions A and B are summarized in Table 3. L* values gradually increased during storage, which may be attributed to surface dehydration and the onset of freezer burns, resulting in a lighter surface appearance. b* values increased from approximately 33 to 36, indicating progressive yellowing, which was likely caused by lipid oxidation and pigment degradation during frozen storage [34]. Under repeated freeze–thaw conditions (condition B), the total color difference (ΔE) followed the order CP > PP > BP, suggesting that BP packaging provided relatively better color stability. Although the differences among packaging types were not statistically significant (*p* > 0.05), PP tended to show slightly higher b* and E values under continuous freezing (condition A), possibly because of its lower gas-barrier efficiency, which may have facilitated mild oxidative discoloration [35]. Overall, no significant differences in color parameters were observed between packaging materials or storage conditions. The observed color changes were primarily attributed to surface dehydration and oxidative reactions during frozen storage. BP consistently exhibited the greatest color stability, particularly under freeze–thaw stress, although variability among treatments was not statistically significant.

#### 3.2.4. Texture Profile Analysis

Texture profile analysis was conducted to evaluate the hardness, springiness, gumminess, and chewiness of frozen fried chicken (Table 4). Texture profile analysis (TPA) was performed exclusively on the surface layer (fried crust/skin) of the frozen fried chicken samples; the underlying muscle tissue was not analyzed separately. This region was selected because it is most directly affected by dehydration and freeze–thaw-induced structural changes during frozen storage and plays a critical role in consumer perception of product quality. Intact surface portions containing the fried crust were analyzed without physically separating the skin from the underlying muscle tissue in order to maintain sample integrity and reproducibility. Samples were tested using a flat cylindrical probe under a two-cycle compression test, and the compression ratio was deliberately selected to primarily deform the surface layer rather than the inner muscle tissue. This testing configuration emphasized surface-related textural parameters, including hardness, springiness, gumminess, and chewiness, and enabled consistent comparison of packaging effects on surface texture stability during frozen storage.

Fried chicken samples packaged in BP under condition A and CP under condition B demonstrated superior structural stability throughout the storage period. Overall, hardness decreased and chewiness increased over time. Repeated freeze–thaw cycling (condition B) caused noticeable softening, followed by a slight recovery, likely owing to tissue reorganization. Springiness was lowest for PP under condition B; however, the difference between PP and BP was not statistically significant (*p* > 0.05). BP and PP maintained food textural integrity comparable to or better than that of conventional CP packaging under both storage conditions. These findings highlight the potential of BP and PP as sustainable alternatives for maintaining the physical quality of frozen ready-to-eat foods during extended storage [36]. The observed textural changes can be directly related to the formation and evolution of ice crystals during frozen storage. Repeated freeze–thaw cycles promote ice crystal growth and recrystallization, which disrupt the microstructure of the fried crust and weaken its mechanical integrity. This structural damage leads to reduced hardness and altered chewiness, as observed under condition B. Packaging systems that effectively limited moisture migration and ice crystal formation, particularly BP, exhibited better preservation of surface texture and showed better preservation of surface texture, indicating that effective moisture regulation and ice crystal control play a critical role in maintaining the textural quality of frozen fried chicken.

## 4. Conclusions

In this study, a sodium alginate (SA)-based biofoam demonstrated strong potential as a sustainable packaging material for frozen foods. The biofoam exhibited a high moisture absorption capacity (>2400%), reflecting its porous and hydrophilic structure, while maintaining adequate structural integrity during frozen storage. Frozen fried chicken packaged with SA-based biofoam showed improved moisture retention, lower lipid oxidation levels (TBARs ranging from approximately 2.5 to 3.6 mg MDA/kg), and enhanced color and surface texture stability compared with conventional plastic and paper packaging, particularly under repeated freeze–thaw conditions over 90 days of storage. Visual evaluation further confirmed reduced ice crystal and frost formation in biofoam-packaged samples, indicating improved moisture regulation and protection against freezer-related quality deterioration. The SA-based biofoam provided physicochemical preservation performance comparable to or superior to that of commercial packaging materials while offering environmental advantages. These findings highlight SA-based biofoam as a promising eco-friendly alternative for cold-chain and frozen food packaging, with future work focusing on scalability, mechanical optimization, and broader applicability across diverse frozen food products.

## Figures and Tables

**Table 1:** Changes in moisture absorption of packaging materials—plastic pouch (CP), paper pouch (PP) and biofoam in a plastic pouch (BP)—after the Gelbo flex test.

Packaging Type	Moisture Absorption (%)	
	Before Gelbo Flex	After Gelbo Flex
Plastic pouch (CP)	0	0
Paper pouch (PP)	3.00 ± 1.42	14.39 ± 4.17
Biofoam in a plastic pouch (BP)	2468.33 ± 28.85	N/A

N/A: not measured because of the inapplicability of the Gelbo flex test pretreatment. Values are expressed as mean ± standard deviation (n = 3).

**Table 2:** Changes in water vapor permeability of packaging materials—plastic pouch (CP) and paper pouch (PP)—during frozen storage.

Packaging Type	WVTR (g/m^2^-Day)	
	Day 0	Day 90
Plastic pouch (CP)	4.25 ± 0.04	9.04 ± 0.03
Paper pouch (PP)	8.08 ± 0.23	20.07 ± 0.27

Note: packaging was pretreated using the Gelbo flex test.

**Table 3:** Color parameters of frozen fried chicken according to packaging type—plastic pouch (CP) and paper pouch (PP), biofoam in a plastic pouch (BP) and frozen storage condition.

Packaging Measure		Storage Period (Days)/Freeze–Thaw Cycles				
		Condition B				Condition A
		0	30 (Cycle 1)	60 (Cycle 2)	90 (Cycle 3)	90 (Cycle 0)
CP	L*	43.39 ± 1.96	46.10 ± 2.00 ^a^	46.73 ± 1.68 ^a^	48.93 ± 2.05 ^a^	45.35 ± 2.17 ^a^
	a*	19.21 ± 1.05	20.11 ± 0.89 ^b^	19.66 ± 0.89 ^a^	20.61 ± 0.96 ^a^	19.67 ± 0.96 ^a^
	b*	33.85 ± 1.46	36.61 ± 1.70 ^a^	36.20 ± 1.15 ^a^	37.46 ± 1.33 ^a^	34.43 ± 1.60 ^a^
	ΔE	-	4.84 ± 3.01 ^b^	5.08 ± 2.49 ^a^	6.76 ± 2.35 ^a^	2.90 ± 1.17 ^a^
PP	L*	43.39 ± 1.96	46.40 ± 1.92 ^a^	47.74 ± 1.58 ^a^	48.07 ± 2.06 ^a^	49.91 ± 1.42 ^b^
	a*	19.21 ± 1.05	20.19 ± 0.92 ^b^	19.94 ± 1.16 ^a^	20.08 ± 0.96 ^a^	19.73 ± 0.82 ^a^
	b*	33.85 ± 1.46	36.28 ± 1.74 ^a^	36.31 ± 1.29 ^a^	36.58 ± 1.08 ^a^	36.58 ± 1.66 ^b^
	ΔE	-	5.20 ± 1.92 ^b^	5.45 ± 2.05 ^a^	5.84 ± 1.61 ^a^	6.53 ± 2.96 ^b^
BP	L*	43.39 ± 1.96	45.65 ± 1.95 ^a^	46.18 ± 1.97 ^a^	46.71 ± 1.32 ^a^	46.07 ± 1.71 ^a^
	a*	19.21 ± 1.05	19.28 ± 0.59 ^a^	19.63 ± 0.83 ^a^	20.29 ± 1.36 ^a^	20.31 ± 1.32 ^a^
	b*	33.85 ± 1.46	35.46 ± 1.43 ^a^	36.04 ± 1.85 ^a^	36.06 ± 1.95 ^a^	36.19 ± 1.40 ^b^
	ΔE	-	2.06 ± 1.05 ^a^	5.13 ± 2.52 ^a^	5.39 ± 2.29 ^a^	3.83 ± 1.89 ^a^

* Values are expressed as mean ± standard deviation. Different superscript letters (a, b) within the same column indicate statistically significant differences (*p* < 0.05), according to one-way ANOVA followed by Duncan’s multiple range test.

**Table 4:** Textural properties of frozen fried chicken according to packaging type plastic pouch (CP) and paper pouch (PP), biofoam in a plastic pouch (BP), and frozen storage condition.

Packaging Parameter		Storage Period (Days)/Freeze–Thaw Cycles				
		Condition B				Condition A
		0	30 (Cycle 1)	60 (Cycle 2)	90 (Cycle 3)	90 (Cycle 0)
CP	Hardness (N)	6.46 ± 0.76	5.68 ± 1.79 ^a^	6.34 ± 1.43 ^a^	5.87 ± 1.23 ^a^	6.63 ± 2.34 ^a^
	Springiness	0.34 ± 0.21	0.34 ± 0.13 ^a^	0.40 ± 0.21 ^a^	0.35 ± 0.20 ^a^	0.52 ± 0.35 ^a^
	Gumminess (N)	2.01 ± 0.58	1.67 ± 0.80 ^a^	3.00 ± 0.57 ^a^	2.31 ± 1.12 ^a^	1.62 ± 0.15 ^a^
	Chewiness (N)	0.46 ± 0.30	0.65 ± 0.42 ^a^	1.65 ± 0.37 ^a^	1.04 ± 0.47 ^a^	0.48 ± 0.13 ^a^
PP	Hardness (N)	6.46 ± 0.76	5.65 ± 1.74 ^a^	6.92 ± 1.45 ^a^	5.64 ± 2.13 ^a^	6.48 ± 1.21 ^a^
	Springiness	0.34 ± 0.21	0.51 ± 0.25 ^a^	0.37 ± 0.16 ^a^	0.24 ± 0.16 ^a^	0.45 ± 0.17 ^a^
	Gumminess (N)	2.01 ± 0.58	2.10 ± 0.62 ^a^	2.15 ± 1.20 ^a^	1.43 ± 0.73 ^a^	2.00 ± 0.84 ^a^
	Chewiness (N)	0.46 ± 0.30	1.05 ± 0.50 ^ab^	1.00 ± 0.50 ^a^	1.09 ± 0.14 ^a^	0.94 ± 0.33 ^a^
BP	Hardness (N)	6.46 ± 0.76	5.86 ± 0.70 ^a^	6.33 ± 1.78 ^a^	5.75 ± 1.80 ^a^	6.75 ± 1.90 ^a^
	Springiness	0.34 ± 0.21	0.49 ± 0.18 ^a^	0.44 ± 0.24 ^a^	0.40 ± 0.18 ^a^	0.30 ± 0.15 ^a^
	Gumminess (N)	2.01 ± 0.58	2.19 ± 0.40 ^a^	2.53 ± 1.03 ^a^	2.05 ± 1.19 ^a^	1.75 ± 0.44 ^a^
	Chewiness (N)	0.46 ± 0.30	1.28 ± 0.60 ^b^	1.33 ± 1.30 ^a^	1.15 ± 0.56 ^a^	0.84 ± 0.07 ^a^

Values are expressed as mean ± standard deviation. Different superscript letters (a, b, and ab) within the same column for the same textural parameter indicate statistically significant differences (*p* < 0.05), according to one-way ANOVA followed by Duncan’s multiple range test.

## Data Availability

The original contributions presented in this study are included in the article. Further inquiries can be directed to the corresponding author.
